# How Chloroform Kills

**Published:** 1859-04

**Authors:** 


*How Chloroform Kills.*—Dr. Chapman, of London, has been writing for the Medical Times and Gazette, on this subject. He believes that the proximate cause of death from chloroform is the mechanical obstruction of the right side of the heart by excessive distention with venous blood. He cites an experiment on a cat, which was caused to inhale chloroform till the action of the heart had ceased. On opening the chest, the heart was quiet and greatly distended; the pericardium embracing it very tightly. As soon as an incision was made through this membrane, the right auricle instantly forced itself through the opening. The pulmonary arteries were then opened, and immediately the heart resumed its action and continued to beat for an hour and a half.

He believes that anæsthesia depends upon the arrest of oxydation in the nervous tissue, a result which may be brought about either by abstraction of oxygen from the corpuscles, which happens when oxydizable substances, such as the vapors of chloroform and ether, are presented to them; or by the induction of oxydation throughout the system, generating great quantities of carbonic acid, and preventing the access of oxygen from without, as in the administration of nitrous oxyd. He thinks that they may kill either by impeding oxydation in the brain, and thus lessening the transmission of influence through the pneumogastric nerve to the heart; by lessening the automatic action of the heart in consequence of diminishing oxydation in its special ganglia; or lastly, by interfering with the circulation through the lungs, and so distending the right side of the heart, as already stated. Of course, persons with feeble hearts can offer less resistance to this exhausting action, and must therefore sooner succumb.

The practical lessons he deduces from this view of the subject, are: to administer the vapor of anæsthetic agents in a state of sufficient dilution with atmospheric air, for which purpose he prefers Dr. Snow's inhaler; to watch the pulse carefully, and to withdraw or dilute the vapor as soon as any stop, flutter, intermission, or even marked retardation or diminished strength is detected; to withdraw the vapor, instead of administering it in fuller doses when the patient begins to struggle, an act which Dr. C. believes to be a true wrestling for life; and lastly, to use artificial respiration, first of pure oxygen and then of atmospheric air, (keeping the body warm meanwhile,) in those cases in which the anæsthetic has produced dangerous symptoms.

